# Instrumental conditioning for food reinforcement in the spontaneously hypertensive rat model of attention deficit hyperactivity disorder

**DOI:** 10.1186/s13104-017-2857-5

**Published:** 2017-10-30

**Authors:** Claire L. Rostron, Victoria Gaeta, Louise R. Brace, Eleanor J. Dommett

**Affiliations:** 1 Dept Life, Health and Chemical Sciences, The Open University, Walton Hall, Milton Keynes, MK7 6AA UK; 2 Department of Psychology, Institute of Psychiatry, Psychology and Neuroscience, King’s College London, Addison House, Guy’s Campus, London, SE1 1UL UK

**Keywords:** Extinction, Reinforcement, Inhibition, Reward, ADHD

## Abstract

**Background:**

The spontaneously hypertensive rat is thought to show good validity as a model of attention deficit hyperactivity disorder, in part because of impaired delayed reinforcement behaviour, corresponding to the dynamic developmental theory of the disorder. However, some previous studies may have been confounded use of fluid reward. Therefore, the objective of this study was to assess the spontaneously hypertensive rat and two comparison strains (Wistar and Wistar Kyoto) using a non-delayed food reinforcement paradigm in an attempt to advance knowledge of basic learnt behaviour in this strain, without potentially confounding reward sensitivity, which could impact on motivation to learn. Rats were trained on a fixed ratio 1 two choice discrimination schedule, extinction, reacquisition and reversal. We also tested non-reinforced spontaneous alternation to facilitate data interpretation.

**Results:**

The spontaneously hypertensive rat displayed slower shaping and reduced on task activity during task acquisition, contrasting with previous results which indicate either enhanced responding and an impairment only when a delay is used; we suggest several reasons for this. In line with previous work, the same strain exhibited poor extinguishing of behaviour but were not impaired to the same extent on reversal of the discrimination. Finally, non-reinforced alternations on a Y-maze were also reduced in the spontaneously hypertensive rat.

**Conclusions:**

In sum, the spontaneously hypertensive rat appear to show poor response inhibition in reinforced and non-reinforced contexts. However, impaired response inhibition was reduced during reversal when an opposite response produced food reward alongside presentation of the conditioned stimulus. We discuss the possibility of enhanced attribution of incentive salience to cues in this strain and highlight several points of caution for researchers conducting behavioural assessments using the spontaneously hypertensive rat and their associated comparison strains.

**Electronic supplementary material:**

The online version of this article (10.1186/s13104-017-2857-5) contains supplementary material, which is available to authorized users.

## Background

Attention deficit hyperactivity disorder (ADHD) affects an estimated 8–12% of children [1] and around 3% of adults [2]. It can be divided into three distinct presentation or subtypes: predominantly inattentive subtype (ADHD-I), predominantly hyperactive/impulsive subtype (ADHD-H), and combined subtype (ADHD-C) [3]. Consequences of the disorder are striking and include poor academic performance, social relationships and a higher risk of drug abuse [4], making it critical that we understand the condition fully. Early research largely focused on executive deficits in ADHD [5] which are thought to be underpinned by alterations to fronto-striatal circuits, including mesocortical dopamine levels [6]. However, several researchers since have proposed alterations to the mesolimbic dopaminergic pathway in ADHD [7–11], as well as changes to other neurotransmitter systems including serotonin [12, 13] and noradrenaline [14, 15]. This wider focus has resulted in particular interest being given to altered reinforcement processing in the condition [16, 17] and the proposed dynamic developmental theory of ADHD, which assumes altered reinforcement sensitivity is a central tenet of the disorder [6].

One way to investigate alterations in processing is to utilize animal models. These offer the potential to study the effects of naturally arising or induced alterations in neurobiology and relate these to behavioural deficits, but only when they are both reliable and valid models of the disorders under scrutiny. The spontaneously hypertensive rat (SHR) is one suggested animal model of ADHD, considered to best model ADHD-C [18], but it is generally accepted to exhibit good validity. In terms of face validity, the strain shows hyperactivity, inattention and impulsivity [6, 19–26]. Moreover these behavioural features alter over the lifespan and occur independently of each other, as is the case in ADHD [6, 27–29]. The SHR has also been found to have neurobiological changes in the dopaminergic and noradrenergic systems, in line with ADHD [30–37]. Furthermore, the neurobiological changes found could support possible reinforcement-related alterations, similar to those seen in ADHD and proposed in the dynamic developmental theory, which posits that altered dopaminergic function plays a pivotal role by failing to modulate non-dopaminergic transmission appropriately [32, 33, 35, 38–44]. Despite good face and construct validity, the SHR as a model of ADHD is not without its criticisms, much of which is because predictive validity of this strain is weak [15, 26, 45, 46]. In addition, the SHR has also been suggested to model some aspects of Schizophrenia [47, 48]. This could suggest that they are not a model of ADHD specifically but rather have alterations to monoamines, and most notably, mesolimbic dopamine levels and attention to reinforcement-related stimuli that could be common to both conditions [49–52]. Despite these limitations the SHR still provides a useful rodent model of ADHD for studies investigating components relating to face or construct validity in the combined type of ADHD.

In line with the utility of the model, much research has been conducted into reinforcement-related behaviour in the SHR, with the view to elucidating such functions in ADHD [29, 53–63]. Whilst some of this work has been conducted with food reinforcement [24, 64–68], a significant proportion has using fluid reward [53–60, 62, 63, 69–71] and it is possible this research may have been confounded by the use of fluid reinforcement. The SHR have altered renal functioning relative to the Wistar Kyoto normotensive strain [72–75] which, we recently showed results in increased fluid intake in the SHR [76]. This means that the SHR are likely to have altered sensitivity to fluid reward, which could impact on motivation to learn. In the current study we therefore assessed performance of the SHR (Charles River, Germany) in comparison to the two different comparison strains on (i) instrumental two choice discriminative conditioning for food on a fixed ratio one schedule advancing through extinction, reacquisition, and reversal; (ii) spontaneous non-reinforced alternation to assess whether the rats exhibited general problems with response inhibition in the absence of reinforcement. We included the normotensive Wistar Kyoto rat (WKY, Charles River, Germany), which does not experience altered renal function and, therefore, fluid reward sensitivity, and the outbred Wistar rat (WIS, Charles River, Germany). It should be noted that whilst these strains serve as a helpful comparison when considering fluid sensitivity, the WKY, from this particular supplier has itself, been suggested as an animal model of the inattentive form of ADHD [18] so cannot be considered as a control for the SHR in terms of ADHD. We draw informative comparisons across tasks that highlight the importance of examining the behavioural characteristics of this strain in greater detail if we are to sensibly judge their use as a model of ADHD and any other a human mental health condition.

## Methods

### Animals

In this study individual rats were classed as experimental units and groups were based on strain, such that there was one experimental group (SHR) and two control groups (WIS and WKY). In total eighteen male rats (SHR N = 6; WKY N = 6; WIS N = 6; Charles River, Germany) aged 10 weeks at the start of testing and group housed throughout were used. This sample size was based our previous work with the three strains [76]. Although ADHD-like behaviours have been found in younger animals [45], this age corresponds with much of the previous work by proponents of the SHR as a model of ADHD [20, 77], including the work done with food reinforcement [65–68]. Upon arrival at the animal unit, rats were housed in standard caging in same-strain pairs, within scantainers with environmental enrichment (tube and bedding). After 2 weeks they were transferred to a reverse dark–light cycle where they remained for the duration of the study. Rats had a further 2 weeks to adjust to the changed cycle before any testing took place. Rats were kept in temperature and humidity controlled rooms, on a reverse dark–light cycle (lights off at 8 a.m. for 12 h). All testing was conducted between 9 a.m. and 4 p.m. and, therefore, in the period when the rats were most active.

All rats were incentivized using a combination of scheduled access to food and food restriction [78] during conditioning. This entailed, each rat being fed 20 g of lab chow per day, at the end of the day’s testing, allowing them to gain some weight each week in line with normal growth for this age range (a growth of between 20 and 40 g, according to growth charts from the supplier, Charles River, Germany). Each rat was weighed daily to ensure healthy weight gain. Whilst this may not induce such a strong motivational drive as traditional food restriction methods where animals are maintained at between 80 and 90% of their free feeding body weight, it is considered more appropriate for group housed animals [79]. Given guidance on food restriction is to ensure the minimum levels of restriction necessary to achieve the scientific objective and to consider the complexity of the behavioural task [78], this combined approach was deemed suitable. Water was freely available throughout. There are known differences in the normal healthy body weights of the three strains employed in the current study, and this was reflected in the starting weight of the rats, prior to habituation, even though they were the same age (WIS = 204 ± 1.8 g; WKY = 188 ± 5 g; SHR = 132 ± 2.5 g). These strain differences were maintained throughout with final weights at task completion also different (WIS = 342 ± 14.5 g; WKY = 337 ± 11.2 g; SHR = 307 ± 13 g). To inform our interpretation of the task data we calculated the percentage increase in body weight normalized to the number of weeks spent completing the task and therefore, being food restricted, and conducted a One-Way ANOVA with post hoc Tukey tests on the data (IBM SPSS Statistical Package Version 23). This showed that the three groups did not have comparable weight gain (F(2, 15) = 14.10, p < 0.001, η^2^ = 0.653) with the WIS (normalized weight gain 8.7%) showing a reduced gain compared to the WKY (normalized weight gain 12.6%, p = 0.004) and SHR (normalized weight gain 14.1% p < 0.001), but no significant difference between the latter two strains. This information is considered in our discussion.

### Food reinforced operant conditioning

Food reinforced operant conditioning was conducted in five-hole wall operant boxes (Med-Associates, St Albans, Vermont, USA) housed within ventilated light/sound insulated chambers in a laboratory adjacent to the animal holding rooms. Only two of the five holes were used, the rest were blocked off. Those used were the immediate left and right of the center hole. The rear wall of the chambers contained a reward port linked to a pellet dispenser delivering 45 mg raspberry flavored reward pellets (Test Diet Precision Pellets, Sandown Scientific, UK). The equipment was controlled by a PC linked to a smart control interface system running Med-PC IV (Med-Associates, St Albans, Vermont, USA). Rats were habituated to the pellets in the home cage in isolation and were required to eat 10 pellets over 2 consecutive days. Shaping began with a free reward stage until at least 30 pellets had been retrieved on each of 2 consecutive days. Rats then progressed to a hopper nose-poke stage where they had to nose-poke into the reward port for delivery of the pellets. Fifty nose-pokes were required on each of 2 consecutive days before conditioning began. Both of these shaping stages had an enforced inter-trial interval (ITI) of 2 s, whilst the conditioning stages were self-paced with a programmed inter-trial interval (ITI) of 0 s. Conditioning began with fixed ratio 1 (FR1) training. FR1 two choice training was conducted until a rat made 50 correct responses on each of 5 consecutive days. What constituted a correct response was counterbalanced by strain such that for half of each strain a correct response was a nose-poke into the left hole and for the remaining animals it was a nose-poke into the right hole. Once 50 correct responses were achieved on each of 5 consecutive days each rat was then advanced onto the extinction schedule for 10 days. The extinction procedure was structured so that a nose-poke still produced onset of the reward port light and a reward port response was still required but no pellet was made available. This approach ensured that sensory cues and motor requirements were identical to FR1 in all regards but the reward availability [80, 81], which is particularly important in SHR which have altered sensory and motor processing [82, 83]. Rats were then tested for reacquisition of FR1 until 50 correct responses were made on each of 2 consecutive days. Reversal testing then began such that each rat was reinforced for a nose-poke into the opposite hole from that which was reinforced in the initial FR1 and reacquisition stages. Reversal continued until a rat made 50 correct nose-pokes and fewer than 10 incorrect nose-pokes over 5 consecutive days. Time out periods of 4 s with the house light on were imposed for all responses other than correct responses throughout.

At all stages, except during the extinction schedule where duration was set to 10 days, the number of days a rat took to reach criterion was recorded. In addition, the following measures were recorded for the different stages of conditioning (FR1, extinction, reacquisition, reversal): percent correct responses, percent incorrect responses, percent late responses (into the correct nose-poke hole once the reward was available for collection), nose-poke discrimination [(correct/incorrect + correct) × 100], percent anticipatory responses (into the reward port when the nose-poke stimulus lights were on) and total on task responses (the sum of correct, incorrect, anticipatory and late responses). Reaction times (RT) and pellet collection times (PT) were also collected for correct responses.

All data were statistically analysed using SPSS (IBM SPSS Statistical Package Version 23). Initially, data were checked for normality using the using the Kolmogorov–Smirnov test. All performance data (days to criterion, response numbers and percentage) were deemed suitable for use with parametric tests. The days to criterion data was analyzed with a One-Way ANOVA, followed by post hoc Tukey tests, as appropriate. All remaining performance variables were then analyzed with repeated measures ANOVA with DAY (on each stage) as the within-subjects variable and STRAIN as the between-subjects variable. Within-subjects difference contrasts were used to elucidate changes between consecutive sessions and Tukey tests were used for post hoc strain comparisons. Data were checked for homogeneity and, where this was violated, the Greenhouse–Geisser correction was employed and corrected degrees of freedom are reported where this was the case. The timing data (RT and PT) were skewed and, therefore, log_10_ transformed to obtain a normal distribution suitable for analysis with parametric tests, although absolute data is shown in the figures to illustrate raw response times. Once transformed this data was analysed in the same manner as the performance variables. Our interpretation of the main data analyses was informed by observed power measures, obtained as part of the ANOVAs.

For the initial FR1 acquisition the first 7 days were analysed, which corresponds to the minimum period any animal took to complete this stage and the period of most intense learning. All 10 days on extinction were analysed. The first 2 days of reacquisition and the first 5 days on reversal were analysed for group differences, again because this was the minimum period necessary to complete the task. By analysing these specific phases in detail as well as noting total days to criterion we conducted the inferential statistics when all animals were on a specific stage, therefore maximising statistical power, whilst also analysing the overall time taken on specific stages.

### Spontaneous non-reinforced alternation

In order to aid interpretation of the non-delayed reinforcement paradigm described above we also conducted test of spontaneous non-reinforced alternation in a second laboratory space in close proximity to the holding room. An eight arm grey plastic radial maze was used with only three arms open reflecting a Y-maze layout. This was situated in a dark, quiet room with extra maze visual cues. The maze was wiped with alcohol after each rat to remove odor cues. Rats were randomly ordered for testing in a single session. They were placed into the centre section and arm entries, defined as when a rat had all four paws within an arm, were recorded for 5 min by an experimenter directly onto a computer using a specially written Python program.

Four parameters were measured for analysis: the total number of arm entries made; number of arm alternations (defined as entry into all three arms consecutively); arm discrimination index (the number of alternations as a proportion of overall entries) and latency to visit the first arm. As with the conditioning task, these data were all analysed using SPSS (IBM SPSS Statistical Package Version 23), beginning by checking for normal distribution using the Kolmogorov–Smirnov test. The total number of arm entries and the latency to first arm were normally distributed and therefore analysed using a One-Way ANOVA and post hoc Tukey tests to determine any strain differences in the absence of any transformations. However, the number of alternations and the discrimination index were not normally distributed and the distribution could not be normalised by log10, arcsin, square root or reciprocal transformations and therefore a non-parametric, Kruskal–Wallis test with post hoc Mann–Whitney analysis was used to determine any strain differences. Post hoc power analysis was also performed on this data.

## Results

### Behaviour of SHR is slower to shape and they show reduced task activity and increased latency during FRI acquisition

#### Habituation and shaping

All rats successfully completed the habituation prior to shaping on the task and there was no significant difference in the number of sessions needed to do this between the three strains, as assessed by a One-Way ANOVA (F(2, 15) = 2.786, p = 0.094, η^2^ = 0.270). All WKY and WIS achieved criterion on each of the two shaping stages over 2 consecutive days. By contrast the SHR took 3.00 ± 0.37 days (mean ± SEM) on the free reward stage, and 4.00 ± 0.73 days (mean ± SEM) on the hopper nose-poke stage. This resulted in significant strain differences for the free reward stage (F(2, 15) = 7.50, p = 0.006, η^2^ = 0.500) and the hopper nose-poke stage (F(2, 15) = 7.50, p = 0.006, η^2^ = 0.500). In both cases the SHR took significantly longer than both other strains (p = 0.011) but there was no significant difference between the WIS and WKY.

#### Acquisition

**Fig. 1:**
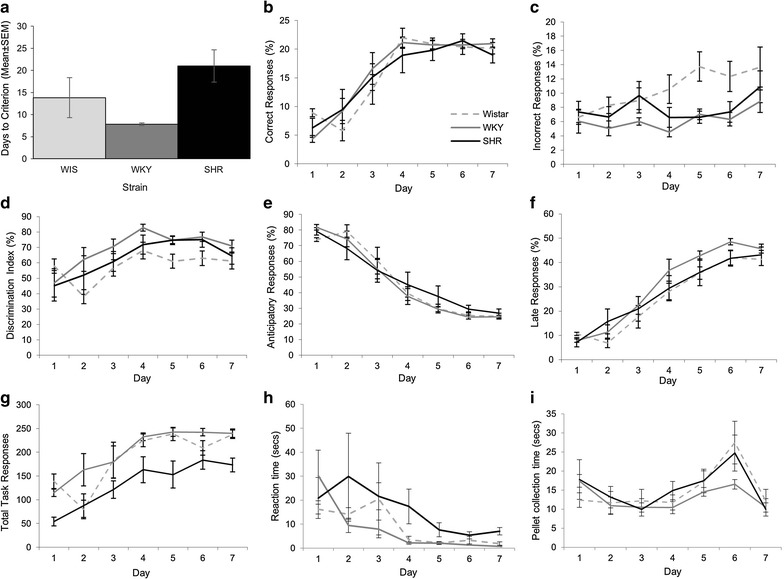
FR1 acquisition. There was no significant difference between strains in the days taken to reach criterion on this task (**a**). The SHR did not differ from the two comparison strains in terms of correct responses (**b**), incorrect responses (**c**), discrimination index (**d**), anticipatory responses (**e**) or late responses (**f**). However, they did show a decrease in total task activity relatively to both other strains (**g**) and increased reaction time (**h**) but not pellet collection time (**i**). Representative key shown in **b**

Although the WKY completed this stage quicker than the other two strains (Fig. 1a), there was no significant strain differences in the number of days taken to reach criterion on this task (F(2, 15) = 3.37, p = 0.062, η^2^ = 0.310). Given that the earliest stage at which criterion was reached was on day 7, we focused our whole group analysis of response parameters on the first 7 days of acquisition allowing all animals to be included for all time points, thus maximizing sample size and statistical power. This analysis showed, as expected, a main effect of DAY for all measures with task performance and engagement generally increasing throughout (Fig. 1b–g: percent correct F(3.12, 46.77) = 37.84, p < 0.001, η_p_^2^ = 0.716; percent incorrect F(3.22, 48.30) = 3.75, p = 0.015, η_p_^2^ = 0.200; nose-poke discrimination F(2.73, 41.05) = 7.49, p < 0.001, η_p_^2^ = 0.333; percent anticipatory (F(2.86, 42.87) = 103.58, p < 0.001, η_p_^2^ = 0.874); percent late (F(2.718, 40.767) = 78.695, p < 0.001, η_p_^2^ = 0.840) and total on task activity (F(3.45, 51.82) = 21.81, p < 0.001, η_p_^2^ = 0.592). For the all measures, within-subject difference contrasts showed no significant differences between day 1 and day 2 followed by significant changes indicating improved performance with days on task, as expected (see Additional file 1: Table S1).

For all measures there was no significant DAY × STRAIN interaction, indicating that the rate of learning was comparable across strains [percent correct F (6.24, 46.77) = 0.88, p = 0.517, η_p_^2^ = 0.105; percentage incorrect F(6.424, 48.30) = 1.21, p = 0.32, η_p_^2^ = 0.139; percent anticipatory F(5.72, 42.87) = 1.13, p = 0.359, η_p_^2^ = 0.131] percent late (F(5.44, 40.77) = 0.918, p = 0.485, η_p_^2^ = 0.109); nose-poke discrimination index (F(5.48, 41.05) = 0.97, p = 0.452, η_p_^2^ = 0.115); total on task activity (F(6.91, 51.82) = 1.19; p = 0.32, η_p_^2^ = 0.138). There was no main effect of STRAIN for percent correct (F(2, 15) = 0.13, p = 0.88, η_p_^2^ = 0.017); however, there was a main effect for percent incorrect (F(2, 15) = 6.28, p = 0.010, η_p_^2^ = 0.455) with the WIS making more incorrect responses compared to WKY (p = 0.008). There was also a significant main effect of STRAIN on nose-poke discrimination index (F(2, 15) = 8.81, p = 0.003, η_p_^2^ = 0.540) with post hoc Tukey tests showing the strain difference was between WIS and WKY only (p = 0.002) with the WIS having a lower discrimination index. There was no significant main effect of STRAIN for late (F(2, 15) = 1.23, p = 0.321, η_p_^2^ = 0.141) or anticipatory responses (F(2, 15) = 0.097, p = 0.908, η_p_^2^ = 0.013). Finally there was a significant main effect of STRAIN for total on task activity (F(2, 15) = 11.71, p = 0.001, η_p_^2^ = 0.610) with post hoc Tukey tests revealing that the SHR made fewer total responses than both the WIS (p = 0.007) and WKY (p = 0.001).

For the latency data (Fig. 1h–i), there was also a significant main effect of DAY [reaction time, RT, F(6, 90) = 23.73, p < 0.001, η_p_^2^ = 0.613; pellet time, PT, F(6, 90) = 7.30, p < 0.001, η_p_^2^ = 0.327]. For RT, latency significantly decreased from day 3 onwards. The pattern across consecutive days was less clear cut for PT (see Additional file 1: Table S1). There was a significant main effect of STRAIN for RT (F(2, 15) = 7.57, p = 0.005, η_p_^2^ = 0.502) with the SHR having an increased reaction time in comparison to both the WKY (p = 0.005) and WIS (p = 0.048). There was also a significant DAY × STRAIN interaction for RT (F6, 90) = 2.08, p = 0.026, η_p_^2^ = 0.217). Within-subjects difference contrasts suggest that this interaction effect is due to the widening of the gap between the SHR and the two comparison strains between day 6 and 7 (p = 0.008). This is confirmed by repeating the analysis excluding day 7. This removes the interaction effect but the main effects of both DAY and STRAIN remain. For PT, there was no significant main effect of STRAIN (F(2, 15) = 1.41, p = 0.274, η_p_^2^ = 0.159) or DAY × STRAIN interaction (F(12, 90) = 0.571, p = 0.860, η_p_^2^ = 0.071).

### SHR exhibit poor extinction of FR1 conditioned responses

**Fig. 2:**
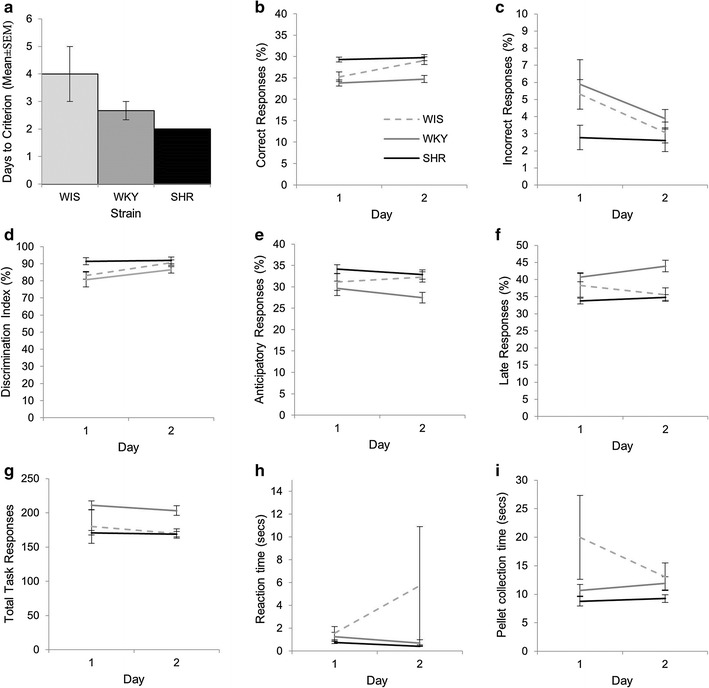
Extinction of FR1. The SHR made more correct responses than both comparison strains (**a**), and fewer incorrect responses than the WKY (**b**), giving a higher discrimination index in the SHR (**c**). There were no strain differences for anticipatory responses (**d**), late responses (**e**), total on task activity (**f**), reaction time (**g**) or pellet collection time (**h**). Representative key shown in **a**

As with FR1 acquisition, there was a significant main effect of DAY for all parameters (Fig. 2a–f): percent correct F(4.75, 71.25) = 13.05, p < 0.001, η_p_^2^ = 0.465; percent incorrect F(4.31, 64.67) = 2.48, p = 0.048, η_p_^2^ = 0.142; nose-poke discrimination F(9, 135) = 6.17, p < 0.001, η_p_^2^ = 0.291; percent anticipatory F(9, 135) = 12.63, p < 0.001, η_p_^2^ = 0.457; percent late F(9, 135) = 12.49, p < 0.001, η_p_^2^ = 0.454 and total on task activity F(4.67, 69.98) = 19.03, p < 0.001, η_p_^2^ = 0.559. The pattern of changes was less consistent than that found for acquisition but generally all measures showed a gradual decrease in task engagement and performance, as would be expected when reinforcement is no longer provided (see Additional file 2: Table S2).

All strains decreased their responding at a comparable rate during the 10 day extinction period as indicated by the lack of a significant DAY × STRAIN interaction for all parameters [percent correct F(9.50, 71.25) = 1.51, p = 0.156, η_p_^2^ = 0.168; percent incorrect F(8.62, 64.67) = 0.70, p = 0.702, η_p_^2^ = 0.085; nose-poke discrimination F(18, 135) = 1.14, p = 0.326, η_p_^2^ = 0.131; percent anticipatory F(18, 135) = 1.014, p = 0.488, η_p_^2^ = 0.119; percent late F(18, 135) = 0.88, p = 0.606, η_p_^2^ = 0.105 and total on task activity F(9.33, 69.98) = 1.70, p = 0.103, η_p_^2^ = 0.185]. In addition, during the 10 day period there was a main effect of STRAIN for percent correct (F(2, 15) = 5.70, p = 0.014, η_p_^2^ = 0.432) with post hoc Tukey tests showing the SHR made more correct responses than both the WKY (p = 0.017) and WIS (p = 0.0465). There was also a main effect of STRAIN for percent incorrect (F(2, 15) = 6.86, p = 0.008, η_p_^2^ = 0.478). For this parameter the only difference was between the SHR and the WKY (p = 0.006) with the SHR showing fewer incorrect responses. In line with the differences in correct and incorrect responses, there was a significant main effect of STRAIN for the nose-poke discrimination (F(2, 15) = 29.45, p < 0.001, η_p_^2^ = 0.797). Post hoc Tukey tests revealed the SHR had a higher discrimination index than both WIS (p < 0.001) and WKY (p < 0.001). There was no main effect of STRAIN on percent anticipatory (F(2, 15) = 1.68), p = 0.220, η_p_^2^ = 0.183), percent late (F(2, 15) = 1.77, p = 0.205, η_p_^2^ = 0.191) or total on task activity (F(2, 15) = 1.28, p = 0.308, η_p_^2^ = 0.145).

Taken together, these results could suggest that the SHR are slower to extinguish their behaviour, however, it should be noted that no strain fully extinguished the behaviour during this period and, therefore, these finding do not reflect extinction as such but rather could reflect a resistance to extinction. To further support this view we conducted an additional analysis using relative data for all response parameters, whereby the responses during extinction were expressed as a percentage of the responses made on the final day of FR1 acquisition. As with our original analysis, there was a significant main effect of DAY for: percent correct F(4.33, 64.91) = 12.92, p < 0.001, η_p_^2^ = 0.463; nose-poke discrimination F(9, 135) = 5.75, p < 0.001, η_p_^2^ = 0.277; percent anticipatory F(4.73, 70.92) = 12.11, p < 0.001, η_p_^2^ = 0.447; percent late F(9, 135) = 13.67, p < 0.001, η_p_^2^ = 0.477 and total on task activity (F(4.93, 73.92) = 20.17, p < 0.001, η_p_^2^ = 0.573). The only parameter no longer showing a main effect of DAY was the percentage of incorrect responses (F(2.73, 41.02) = 2.56, p = 0.073, η_p_^2^ = 0.146). For those parameters showing a significant main effect, the pattern was consistent with our original analysis showing a gradual decrease in task engagement and performance, as would be expected when reinforcement is no longer provided. Again, as with our original analysis, all strains decreased their responding at a comparable rate during the 10 day extinction period as indicated by the lack of a significant DAY × STRAIN interaction for all parameters [percent correct F(8.65, 64.91) = 1.91, p = 0.068, η_p_^2^ = 0.203; percent incorrect F(5.469, 41.02) = 0.49, p = 0.960, η_p_^2^ = 0.061; nose-poke discrimination F(18, 135) = 1.14, p = 0.319, η_p_^2^ = 0.132; percent anticipatory F(19.46, 70,392) = 1.01, p = 0.374, η_p_^2^ = 0.128; percent late F(18, 135) = 0.86, p = 0.623, η_p_^2^ = 0.103 and total on task activity F(4.93, 73.92) = 1.74, p = 0.089, η_p_^2^ = 0.188].

The new analysis did reveal slightly different strain differences during the 10 day period; there was no main effect of STRAIN for percent correct (F(2, 15) = 1.76, p = 0.205, η_p_^2^ = 0.190), percent incorrect (F(2, 15) = 0.184, p = 0.833, η_p_^2^ = 0.024) or total task activity (F(2, 15) = 0.854, p = 0.445, η_p_^2^ = 0.102). However, there was still a significant main effect of STRAIN for the nose-poke discrimination (F(2, 15) = 6.27, p = 0.011, η_p_^2^ = 0.455). Post hoc Tukey tests revealed the SHR had a higher discrimination index than the WKY (p = 0.008). In this analysis there was also a significant main effect of STRAIN on percent anticipatory (F(2, 15) = 6.235, p = 0.011, η_p_^2^ = 0.454) with post hoc Tukey tests showing the WKY make more anticipatory responses than the SHR (p = 0.008). There was also a significant main effect of STRAIN for percent late (F(2, 15) = 8.31, p = 0.004, η_p_^2^ = 0.525) with the SHR making more late responses than the WKY (p = 0.003). These results, notably the increased nose-poke discrimination, again suggest that the SHR are showing some resistance to extinction.

For the latency data (Fig. 2g–h), there was a main effect of DAY for RT (F(9, 135) = 4.88, p < 0.001, η_p_^2^ = 0.245) but not PT (F(9, 135) = 1.67, p = 0.102, η_p_^2^ = 0.100). For RT within-subject difference contrasts showed that reaction time generally increased with time on task as would be expected when the reward is no longer available (see Additional file 2: Table S2). There was no main effect of STRAIN for RT (F(2, 15) = 0.609, p = 0.557, η_p_^2^ = 0.075) or PT (F(2, 15) = 0.32, p = 0.733, η_p_^2^ = 0.041). There was also no significant DAY × STRAIN interaction for RT (F(18, 135) = 0.862, p = 0.625, η_p_^2^ = 0.103) or PT (F(18, 135) = 1.31, p = 0.190, η_p_^2^ = 0.149).

### SHR may show better FR1 reacquisition

**Fig. 3:**
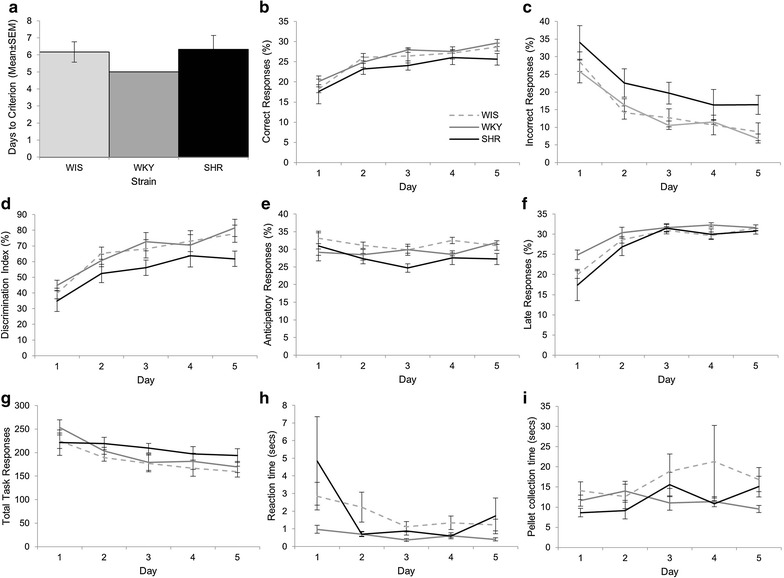
Reacquisition of FR1. There was no significant difference between the strains in the number of days required to reach criterion on this stage (note that all SHR completed in 2 days and therefore the SEM = 0) (**a**). SHR did show an increased percentage of correct responses (**b**) but no difference in incorrect responses (**c**). However, the increase in correct responses was sufficient to result in a lower discrimination index in the SHR (**d**). There were also significant differences between the SHR and comparison strains for anticipatory responses (**e**), late responses (**f**) and total task activity (**g**). There were no strain differences for RT (**h**) but SHR did have reduce pellet collection times relative to the WIS (**i**). Representative key shown in **b**

All strains took a similar time to complete reacquisition (Fig. 3a) and there was no significant difference between strains in the number of days taken to reach criterion on this stage (F(2, 15) = 2.80, p = 0.093, η^2^ = 0.272). For the response parameters during reacquisition (Fig. 3b–g) there was a significant main effect of DAY for percent correct (F(1, 15) = 8.44, p = 0.011, η_p_^2^ = 0.359), percent incorrect (F(1, 15) = 4.79, p = 0.045, η_p_^2^ = 0.242) and nose-poke discrimination (F(1, 15) = 5.12, p = 0.039, η_p_^2^ = 0.255). Between day 1 and day 2 on reacquisition the number of correct responses increased and incorrect responses decreased leading to an increase in discrimination index. There was no significant effect of DAY for percent anticipatory (F(1, 15) = 0.693, p = 0.418, η_p_^2^ = 0.044), percent late (F(1, 15) = 0.224, p = 0.643, η_p_^2^ = 0.015) and total on task activity (F(1, 15) = 0.70, p = 0.416, η_p_^2^ = 0.045). There was no significant DAY × STRAIN interaction for any of the parameters measured [percent correct F(2, 15) = 3.19, p = 0.07, η_p_^2^ = 0.298; percent incorrect F(2, 15) = 0.95, p = 0.411, η_p_^2^ = 0.112; nose-poke discrimination F(2, 15) = 1.07, p = 0.368, η_p_^2^ = 0.125; percent anticipatory F(2, 15) = 1.215, p = 0.324, η_p_^2^ = 0.139; percent late F(2, 15) = 2.54, p = 0.113, η_p_^2^ = 0.253 and total on task activity F(2, 15) = 0.09, p = 0.915, η_p_^2^ = 0.012]. Significant main effects of STRAIN were found for percent correct (F(2, 15) = 16.24, p < 0.001, η_p_^2^ = 0.684) with post hoc Tukey tests showing SHR gave significantly more correct responses in contrast to the WKY (p < 0.001) but not the WIS (p = 0.053). There was no significant main effect of STRAIN for percent incorrect (F(2, 15) = 3.10, p = 0.075, η_p_^2^ = 0.293), but there was a significant main effect of STRAIN for nose-poke discrimination (F(2, 15) = 5.10, p = 0.020, η_p_^2^ = 0.405) with post hoc Tukey tests showing that SHR had higher discrimination than the WKY (p = 0.016) only in their discrimination. There was also a significant main effect of STRAIN for percent anticipatory (F(2, 15) = 4.68, p = 0.026, η_p_^2^ = 0.384) with SHR making more anticipatory responses that the WKY only (p = 0.022). The reverse was found for late responses (F(2, 15) = 6.173, p = 0.011, η_p_^2^ = 0.451) where post hoc Tukey tests found that SHR made significantly fewer late responses in comparison to the WKY (p = 0.009) only. Total on task activity was also significantly different between the strains (F(2, 15) = 5.15, p = 0.020, η_p_^2^ = 0.407) with post hoc tests revealing the WKY to have elevated levels of responding compared the SHR (p = 0.025), and a trend towards the same result with the WIS (p = 0.054). Repeating the ANOVA with the final day of extinction performance as a fixed covariate in order to take account the increased resistance to extinction in the SHR reported above, had little impact on these main effects of STRAIN with all remaining significant as reported with the exception of the discrimination index. This indicates that the altered reacquisition performance of the SHR may not be entirely due to the increased resistance to extinction.

To further examine this and given that relative data was used to support the resistance to extinction, we also conducted an additional analysis on reacquisition data, normalising the response to the final day of extinction. The main effects of DAY were as reported for our initial analysis described above; for percent correct (F(1, 15) = 7.05, p = 0.018, η_p_^2^ = 0.320), percent incorrect (F(1, 15) = 5.87, p = 0.031, η_p_^2^ = 0.311) and nose-poke discrimination (F(1, 15) = 4.98, p = 0.041, η_p_^2^ = 0.249) there was a significant main effect with the number of correct responses increasing and incorrect responses decreasing between days 1 and 2, leading to an increase in discrimination index. There also still was no significant effect of DAY for percent anticipatory (F(1, 15) = 1.17, p = 0.297, η_p_^2^ = 0.072), percent late (F(1, 15) = 0.036, p = 0.852, η_p_^2^ = 0.002) and total on task activity (F(1, 15) = 0.44, p = 0.516, η_p_^2^ = 0.029). Again, in line with our initial analysis, there was no significant DAY × STRAIN interaction for percent incorrect F(2, 15) = 0.42, p = 0.788, η_p_^2^ = 0.036; nose-poke discrimination F(2, 15) = 1.21, p = 0.325, η_p_^2^ = 0.139; percent anticipatory F(2, 15) = 1.00, p = 0.391, η_p_^2^ = 0.118; percent late F(2, 15) = 2.46, p = 0.119, η_p_^2^ = 0.247 and total on task activity F(2, 15) = 0.07, p = 0.936, η_p_^2^ = 0.009). However, there was a significant interaction for percent correct (F(2, 15) = 5.93, p = 0.013, η_p_^2^ = 0.441). Examination of the data reveals this is likely to be due to an increase in correct responses for both the WIS and WKY but not the SHR between the 2 days. There was no significant main effect of STRAIN for percent correct (F(2, 15) = 1.58, p = 0.238, η_p_^2^ = 0.174), percent incorrect (F(2, 15) = 0.143, p = 0.868, η_p_^2^ = 0.022), nose-poke discrimination (F(2, 15) = 1.22, p = 0.322, η_p_^2^ = 0.140), late responses (F(2, 15) = 1.41, p = 0.274, η_p_^2^ = 0.159) or total activity (F(2, 15) = 3.63, p = 0.052, η_p_^2^ = 0.326). However, there was a significant main effect of STRAIN for percent anticipatory (F(2, 15) = 4.94, p = 0.022, η_p_^2^ = 0.397) with SHR making more anticipatory responses that the WKY (p = 0.042) and the WIS (p = 0.037). These data suggest a slightly different picture and may indicate that the improved reacquisition is linked to the resistance to extinction, although further studies would likely be needed to elucidate this.

For latency (Fig. 3h–i), there was a significant main effect of DAY for RT (F(1, 15) = 6.60, p = 0.021, η_p_^2^ = 0.306) with a significant increase between the 2 days (p = 0.021). There was no significant main effect of STRAIN (F(2, 15) = 2.84, p = 0.09, η_p_^2^ = 0.275) and no significant DAY × STRAIN interaction for RT (F(2, 15) = 1.31, p = 0.300, η_p_^2^ = 0.148). For PT there was no main effect of DAY (F(1, 15) = 0.004, p = 0.954, η_p_^2^ < 0.001) or an interaction between DAY × STRAIN (F(2, 15) = 0.35, p = 0.71, η_p_^2^ = 0.045) but there was a main effect of STRAIN (F(2, 15) = 4.31), p = 0.033, η_p_^2^ = 0.365) with the SHR having a reduced PT relative to the WIS (p = 0.026).

### Reversal was comparable across strains

**Fig. 4:**
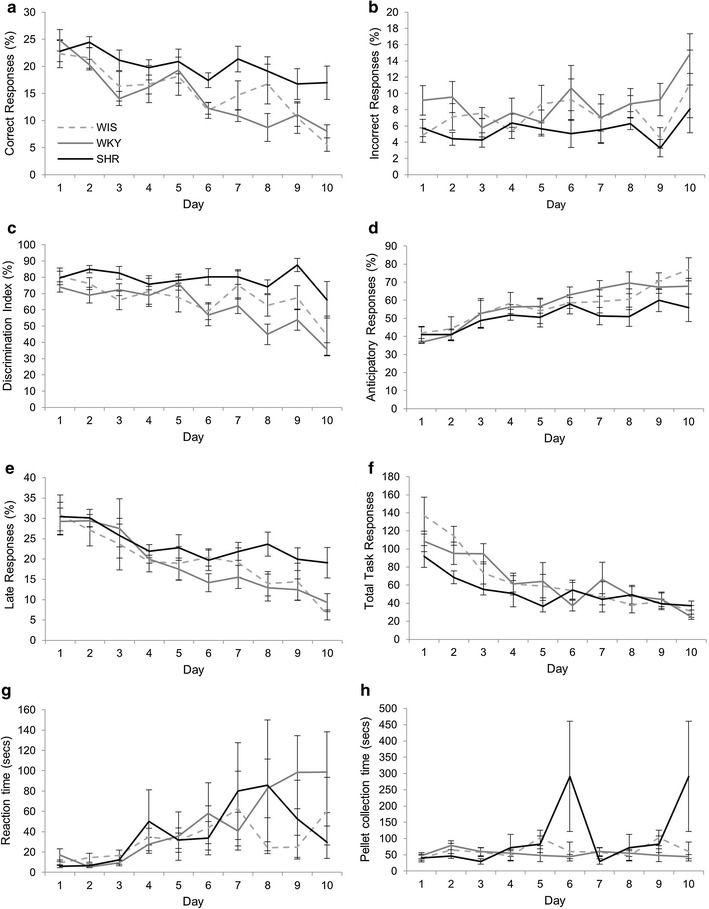
Reversal of the conditioned discrimination in FR1. There was no significant differences between the SHR and comparison strains in the days taken to reach criterion on this task (note that all WKY completed in 5 days and therefore the SEM = 0) (**a**), correct responses (**b**), incorrect responses (**c**), discrimination index (**d**), late responses (**f**), total task activity (**g**) or latencies (**h**, **i**). However, the SHR did make fewer anticipatory responses (**e**). Representative key shown in **b**

All strains took a similar time to complete reversal (Fig. 4a) and there was no significant difference between strains in the number of days taken to reach criterion on this stage (F(2, 15) = 1.58, p = 0.239, η^2^ = 0.174). During reversal there was a significant main effect of DAY for the all parameters except percent anticipatory responses (Fig. 4b–g: percent correct F(2.08, 31.28) = 34.60, p < 0.001, η^2^ = 0.698; percent incorrect F(4, 60) = 39.85, p < 0.001, η^2^ = 0.727; nose-poke discrimination F(4, 60) = 42.38, p < 0.001, η^2^ = 0.739; percent late (F(2.29, 34.38) = 37.206, p < 0.001, η^2^ = 0.713); and total on task activity F(1.80, 27.00) = 11.60, p < 0.001, η^2^ = 0.436; percent anticipatory (F(1.84, 27.57) = 1.60, p = 0.187, η^2^ = 0.096), with performance generally showing slight improvements across time (see Additional file 3: Table S3). There was no significant DAY × STRAIN interaction for any of the parameters measured (percent correct F(4.174, 31.28) = 0.56, p = 0.70, η^2^ = 0.07; percent incorrect F(8, 60) = 0.41, p = 0.913, η^2^ = 0.051; nose-poke discrimination F(8, 60) = 0.928, p = 0.501, η^2^ = 0.110; percent anticipatory F(3.58, 27.57) = 1.07, p = 0.388, η^2^ = 0.125; percent late F(4.583, 34.38) = 1.40, p = 0.250, η^2^ = 0.158 and total on task activity F(3.60, 27.00) = 1.13, p = 0.36, η^2^ = 0.131). Similarly, there was no main effect of strain for percent correct (F(2, 15) = 1.81, p = 0.197, η^2^ = 0.195), percent incorrect (F(2, 15) = 3.55, p = 0.055, η^2^ = 0.321), nose-poke discrimination (F(2, 15) = 3.12, p = 0.074, η^2^ = 0.294), percent late (F(2, 15) = 2.73, p = 0.097, η^2^ = 0.267) and total on task activity (F(2, 15) = 1.84, p = 0.193, η^2^ = 0.197). However, there was a significant main effect of STRAIN for percent anticipatory (F(2, 15) = 4.64, p = 0.027, η^2^ = 0.382) with the SHR making significantly fewer anticipatory responses than WIS (p = 0.021) only.

Latency data (Fig. 4h–i) showed a main effect of DAY for RT (F(1.99, 8.07) = 5.03, p = 0.001, η^2^ = 0.251) but this was due to small and inconsistent changes in reaction time across days (see Additional file 3: Table S3). There was also a main effect of STRAIN for RT (F(2, 15) = 5.84, p = 0.013, η^2^ = 0.438) with post hoc Tukey tests showing a significant difference only between the WIS and WKY (p = 0.01), with the WKY having smaller reaction times. There was no significant DAY × STRAIN interaction (F(3.98, 8.07) = 0.53, p = 0.714, η^2^ = 0.066) for RT. For PT there was no main effect of DAY (F(4.67, 25.00) = 0.747, p = 0.564, η^2^ = 0.047) or STRAIN (F(2, 15) = 1.20, p = 0.328, η^2^ = 0.138) and no significant DAY × STRAIN interaction (F(3.33, 25.00) = 2.09, p = 0.122, η^2^ = 0.218).

### Non-reinforced spontaneous alternation

The data for each parameter are shown in Table 1. There were no strain differences in the total number of arm responses made (F(2, 15) = 1.99, p = 0.17, η^2^ = 0.21) or the latency to visit the first arm (F(2, 15) = 0.30, p = 0.75, η^2^ = 0.034). However, there were significant differences in the number of alternations (χ^2^(2) = 7.36, p = 0.025, η^2^ = 0.433) and the discrimination index (χ^2^(2) = 6.73, p = 0.035, η^2^ = 0.396). Post hoc Mann–Whitney U tests revealed that in both cases the significant difference arose due to the SHR making significantly fewer alternations (U = 1.50, p = 0.004) and therefore having a lower discrimination index (U = 4.00, p = 0.026) than the WIS.

**Table 1:** Performance on the spontaneous alternation task

	Strain		
	SHR	WKY	WIS
Total number of arm entries (mean ± SEM)	7.50 ± 0.76	10.00 ± 1.29	9.00 ± 0.36
Latency to first arm (s) (mean ± SEM)	12.39 ± 3.21	9.07 ± 3.45	9.53 ± 3.21
Total number of alternations [median (IQR)]	1.00 (1.00–1.25)	2.50 (1.00–3.25)	2.50 (2.00–3.00)
Discrimination index [median (IQR)]	0.13 (0.11–0.25)	0.22 (0.17–0.26)	0.28 (2.40–0.31)

Data for the normally distributed variables are expressed as the mean ± SEM for each of the strains and the non-normally distributed variables are given as median (inter-quartile range)

## Discussion

During initial acquisition of FR1 conditioned responses, the SHR showed slower shaping, substantially reduced on task activity, and slower nose-poke reaction times, but not pellet collection times. The individual performance parameters showed that the SHR did not differ significantly from the comparison strains on any one type of response during acquisition, which could indicate that all types of response contributed to this overall reduction in on task activity, or that another factor played a role. The finding of reduced on task activity is in line with the state regulation model of ADHD [84] and task responding reported in ADHD [85]. However, it is at odds with previous operant studies with the SHR, which commonly find increased task responding [20, 56, 86]. There are a number of possible explanations for this. Firstly, it is possible that the SHR showed reduced overall locomotor activity and this resulted in reduced on task activity. Our previous work with these three strains at comparable ages indicates no differences in locomotor activity [76]. This is supported by the lack of strain differences in total number of arm visits on the spontaneous alternation task in the present study, although statistical power may have been compromised (see later discussion). Taken together the results suggest that the activity change during FRI is not reflective of generalised locomotor depression but a greater sample size and concurrent locomotor testing would establish this more clearly. Secondly, reduced on task activity may have arisen as a result of a stress reaction upon being placed in the operant chamber, that is specific to the SHR. Previous work has indicated that the SHR do display an increased stress response [86]. Whilst this may be expected to result in a reduction in locomotor activity, for example, with rats exhibiting freezing behaviour, studies have shown that the SHR has an active response to stress [87] and altered escape behaviour [88]. Therefore, it is possible that the reduced on task activity is a result of increased escape behaviour in the SHR at being placed in an operant chamber which is effectively a constrained space from which the rat may wish to escape. Such a response would likely reduce with experience in the chamber and therefore could also explain why we and Hand et al. [64] found that the low task engagement by SHR reduces over time. Thirdly, reduced on task activity in the SHR could have arisen through increases in other off task behaviours. Future studies using video data recording of behaviour in the chamber would allow a clearer understanding of the reasons behind the reduced on task behaviour [89]. Fourthly, as discussed in the introduction, many of the previous studies used fluid reward and, in support of this contributing to our findings, work using a similar paradigm to the current study and food reward also found decreased response acquisition in the SHR [64]. Fifthly, it is possible that the strains were differentially motivated due to the differences in weight of these strains and the weight gained during the task. As outlined in the methods the SHR gained significantly more weight than the WIS but not the WKY. Given that the SHR showed reduced responding in comparison to both strains, and not just the WIS, the differences in weight gain and subsequent motivation seem unlikely to fully account for this behaviour. Finally, strain specific flavour preferences may have resulted in the SHR showing a reduced preference for the flavoured pellets used in the current study. Evidence suggests that different strains of rat show distinct flavour preferences [87]. It is, therefore, possible that strain differences found here represent differences in flavour preference only. However, this seems unlikely given that all rats habituated to eat the required number of pellets on 2 consecutive days over a comparable period at the start of training but it cannot be ruled out without the addition of specific flavour preference testing.

If we assume the impaired acquisition on this task to be a genuine result, rather than one induced by motivation, flavour preferences or locomotor difference, it is also notable because it has previously been argued that the SHR is impaired in their engagement with operant tasks, only when the task involves delayed reinforcement [64]. This delay-dependent impairment matches findings for ADHD [16] and ties in with arguments of others about the altered delay of reinforcement gradient within the condition and therefore the validity of the SHR as a model of ADHD [6, 57]. In contrast, the findings from the present study suggest that this impairment is not dependent on delayed reinforcement because here there was no delay in reinforcement (none was programmed and pellet collection time was not different between the strains) and yet we still observed a reduction in on task activity, and therefore engagement. Additionally, as stated above we are able to show that this low task engagement by SHR disappears over time, as observed in the study by Hand and colleagues [64], indicating the low engagement we observed is comparable to previous work in some manner. We propose that the disappearance of the low task engagement is a consequence of increasing experience in the chambers rather than removal of the delay, as has been argued [64]. One explanation for the lack of engagement in the absence of a delay in contrast to previous work is the difference in reinforcers used. Previous work has used fluid reinforcers which may result in differences in reward sensitivity between strains [76]. It is plausible that where no delay is used the SHR appears comparable to other strains for fluid reinforcers but that when a delay is present, the SHR is disadvantaged because of a higher basal level of fluid intake and arguably therefore greater drive to obtain this form of reinforcement.

Despite the overall reduction in on task activity, it is noteworthy that the SHR learned the FR1 schedule at the same rate as the comparison strains (based on the gradient of improvement across sessions) demonstrating the importance of taking into account overall activity levels when interpreting SHR data [45]. The present study adds to our previous work [26] indicating that it is inappropriate for researchers to expect an absolute number of responses from SHR to be the same as for comparison strains, as is typically done in behavioural tasks to establish “criterion” performance.

Although no strain fully extinguished the behaviour across the 10 days, the SHR showed particular resistance to extinction in comparison to the WKY, as evidenced in the analysis using absolute responses and relative responses. Problems extinguishing behaviour in the SHR has been reported previously [55, 64]. Therefore, this finding appears to hold across slightly different behavioural paradigms and reinforcer types. It should be noted, however, that a further complication arises with the WKY comparison strain here because recent evidence suggests that the WKY from Charles River actually ignore relevant cues so extinction could be enhanced in this strain [88]. Examination of the post hoc tests indicate that in all bar one case, the significant difference is between the WKY and SHR, with the WIS falling between the two. This indicates that the apparently enhanced resistance to extinction in the SHR, may not be as enhanced as it appears.

However, if we assume that the SHR do show enhanced resistance to extinction, this needs to be interpreted with the data from the non-reinforced alternation task, which indicates problems with response inhibition in the *absence* of reinforcement. It is possible that this reflects an over-arching deficit in response inhibition, that Barkley has argued forms a core deficit in ADHD [5]. The fact that SHR persisted with nose-poke responding during extinction when the light stimulus was presented in the absence of reward pellets could suggest that SHR have attributed greater incentive salience to the light stimulus. A tendency for increased attribution of incentive salience to reward associated cues has been demonstrated in so called ‘sign-trackers’ [52] and this has been suggested to be indicative of an addictive phenotype [49] which has also been linked to impulsivity [89]. Therefore, whether SHR exhibit sign-tracking specifically in a Pavlovian conditioned association task designed to elicit sign tracking is worthy of future investigation. However, it should not be forgotten that the SHR also exhibit hypertension and several brain changes, such as white matter damage, that are linked to this [90]. Therefore it is plausible that the problems with response inhibition in the SHR could specifically be linked to the hypertensive phenotype of these animals. As we did not specifically measure blood pressure in our animals we recognise this as a possibility, although hypertension worsens in the SHR over time [90] whereas we found evidence of improvements in behavioural performance which may not be consistent with such an explanation for our data. SHR showed a much higher level of correct responses during reacquisition, thus re-acquiring to a greater extent. This is likely to be, at least in part, due to the reduced extinguishing of the behaviour. However, of most interest is the reversal stage where SHR did not show significant problems with learning the reversal, despite the initial FR1 reductions in on task activity. There was a trend for mild persistence with the previously correct response which suggests that SHR coped much better with losing the conditioned association when there was another rewarded association taking its place (as opposed to extinction). During reacquisition SHR showed evidence of enhanced reward focus making fewer late responses at the nose-poke hole and more anticipatory responses at the reward port. This effect was notably absent during extinction which does not match the “frustration” that has been reported in children with ADHD when reward is expected but not present [91], although there are clear differences in study methods limiting our ability to make strong comparisons.

It is noteworthy that the two comparison strains did differ from one another on a number of measures and in some cases the SHR differed from only one of the comparison strains, most commonly the WKY, which may indicate that either one or both of these strains is not a suitable control. The problem of a suitable comparison strain for the SHR is not a new one and has been reported previously [92–95]. Much focus has been placed on abnormalities of the WKY but in the present study the WIS was shown to have unexpectedly poor discrimination of the reinforced nose-poke hole despite time out penalties imposed for incorrect responses. The present study therefore adds to the known data about different comparison strains and consequently the debate into this matter. This study also contributes to the discussion on the validity of the SHR as a rodent model of ADHD. As outlined in the introduction, the SHR has been shown to have good face and construct validity in many different studies using a variety of techniques. The results of the present study support a deficit in reinforcement learning but suggest that these need not be limited to delayed reinforcement and that they could arise from a variety of different factors including stress behaviours and sign-tracking. We also demonstrate that some, but not all alterations appear to be dependent on the modality of the reinforcer.

Finally, it is important to note that we collected our data using strain group sizes of N = 6. We therefore examined observed power for the conditioning task and actual effect sizes with post hoc power calculations for the spontaneous alternation task. These calculations demonstrated that our main conclusions are supported by analyses with sufficient power. For the conditioning data all analyses of the main effects of DAY for FR1 acquisition and extinction data had an observed power exceeding the accepted level of statistical power (0.80). This was also the case for all measures on reversal training except anticipatory responses. Therefore, it is possible that a lack of power may explain the lack of significant effects for this measure. For the reacquisition training the majority of measures did not reach the required power standard for main effects of DAY and therefore a greater sample size may increase the number of significant main effects of DAY on this measure. For main effects of STRAIN, which were arguably the focus of this work, observed power varied more than for DAY. In the FRI analysis power exceeded the standard level for total responses, incorrect responses and discrimination index and neared the accepted level for pellet collection latency. All of these showed significant strain differences. For other measures the power was lower and results were non-significant indicating that further strain differences may be found with a larger sample size. A similar pattern was found for extinction data with high power for correct and incorrect responses and discrimination, all of which were significant. Similarly for reacquisition data, high power was associated with significant results for total responses, correct, anticipatory and late responses, indicating other measures may show significant findings if sample size, and therefore power, is increased in future work. The same was true for reversal training with only one measure (incorrect responses) showing near standard levels of power and consequently a significant result. For the spontaneous alternation task, effect sizes were large (f = 0.49–0.89 where an effect size of over 0.4 is consider large for this measure of effect size) for all measures collected except latency where the effect size would be considered small (f = 0.186). For the largest effect size, found for the discrimination index, the accepted level of statistical power (0.80) was exceeded (0.88) with our sample size. For the remaining measures power was lower meaning an increased risk of Type II error. However, examination of the results reported indicate that this is likely to have effected latency and arm entries only because there was a significant strain difference for the number of alternations. Therefore, for the spontaneous alternation task, whilst low statistical power may have impacted on some measures it would not explain the results found including the significant strain differences. In summary, statistical power limitations did likely impact on the current work but, they do not detract from the significant results and our conclusions indicating that modality of reinforcement is important when conducting behavioural tasks with this strain.

## Conclusions

In conclusion, we have highlighted significant issues with working with the SHR strain and its associated comparison strains in behavioural research. We do not, however, wish to criticise the use of animal models of human mental health disorders as these can have exceptional utility for linking brain disturbances to behavioural dysfunction. Rather we wish to emphasize the need for careful and detailed behavioural profiling of animal models, and in this respect argue that much work remains to be done with the SHR and comparison strains.

## Additional files

**Additional file 1: Table S1.** Within-subject contrasts for task acquisition. Summary of within-subject contrasts for the significant main effect of day during task acquisition, showing comparisons to the previous day. α indicates a significant increase from the day before whilst β indicates a significant decrease.

**Additional file 2: Table S2.** Within-subject contrasts for task extinction. Summary of within-subject contrasts for the significant main effect of day during task extinction, showing comparisons to the previous day. α indicates a significant increase from the day before whilst β indicates a significant decrease.

**Additional file 3: Table S3.** Within-subject contrasts for task reversal. Summary of within-subject contrasts for the significant main effect of day during task reversal, showing comparisons to the previous day. α indicates a significant increase from the day before whilst β indicates a significant decrease.

## Abbreviations

ADHD: attention deficit hyperactivity disorder
ADHD-C: combined subtype
FR1: fixed ratio 1
ITI: inter-trial interval
PT: pellet collection times
ADHD-H: predominantly hyperactive/impulsive subtype
ADHD-I: predominantly inattentive subtype
RT: reaction times
SHR: spontaneously hypertensive rat
WKY: Wistar Kyoto rat
WIS: Wistar rat
