# Supplementary material for: Instrumental conditioning for food reinforcement in the spontaneously hypertensive rat model of attention deficit hyperactivity disorder
Source: BMC Res Notes. 2017 Oct 30;10:525. doi: 10.1186/s13104-017-2857-5 (PMC5661932; doi:10.1186/s13104-017-2857-5)
Supplement: Supplementary file 1 — Additional file 1: Table S1. Within-subject contrasts for task acquisition. Summary of within-subject contrasts for the significant main effect of day during task acquisition, showing comparisons to the previous day. α indicates a significant increase from the day before whilst β indicates a significant decrease. [file 13104_2017_2857_MOESM1_ESM.docx]

| Measure | Day 1-2 | Day 2-3 | Day 3-4 | Day 4-5 | Day 5-6 | Day 6-7 |
| --- | --- | --- | --- | --- | --- | --- |
| Percent correct | p=0.214 | p<0.001^α^ | p<0.001 ^α^ | p<0.001^β^ | p<0.001^α^ | p<0.001^β^ |
| Percent incorrect | p=0.992 | p=0.223 | p=0.968 | p=0.014^α^ | p=0.166 | p=0.009^α^ |
| Percent anticipatory | p=0.140 | p<0.001^β^ | p<0.001^β^ | p<0.001^β^ | p<0.001^β^ | p<0.001^β^ |
| Percent late | p=0.118 | p<0.001^α^ | p<0.001^α^ | p<0.001^α^ | p<0.001^α^ | p<0.001^α^ |
| Nose-poke discrimination | p=0.904 | p=0.045^α^ | p=0.001^α^ | p=0.001^β^ | p=0.005^α^ | P=0.446 |
| Total number of responses | p=0.669 | p=0.009^α^ | p<0.001^α^ | p<0.001^α^ | p<0.001^α^ | p<0.001^α^ |
| Reaction Time (RT) | p=0.189 | p=0.165 | p<0.001^β^ | p<0.001^β^ | p<0.001^β^ | p<0.001^β^ |
| Pellet Time (PT) | p=0.078 | p=0.126 | p<0.980 | P=0.020^α^ | p<0.001^α^ | P=0.013^α^ |

Table S1: Summary of within-subject contrasts for the significant main effect of day during task acquisition, showing comparisons to the previous day. ^α^ Indicates a significant increase from the day before whilst ^β^ indicates a significant decrease.
