# Supplementary material for: Instrumental conditioning for food reinforcement in the spontaneously hypertensive rat model of attention deficit hyperactivity disorder
Source: BMC Res Notes. 2017 Oct 30;10:525. doi: 10.1186/s13104-017-2857-5 (PMC5661932; doi:10.1186/s13104-017-2857-5)
Supplement: Supplementary file 2 — Additional file 2: Table S2. Within-subject contrasts for task extinction. Summary of within-subject contrasts for the significant main effect of day during task extinction, showing comparisons to the previous day. α indicates a significant increase from the day before whilst β indicates a significant decrease. [file 13104_2017_2857_MOESM2_ESM.docx]

| Measure | Day 1-2 | Day 2-3 | Day 3-4 | Day 4-5 | Day 5-6 | Day 6-7 | Day 7-8 | Day 8-9 | Day 9-10 |
| --- | --- | --- | --- | --- | --- | --- | --- | --- | --- |
| Percent correct | p=0.415 | p<0.001^β^ | p=0.013^α^ | p=0.665 | p<0.001^β^ | p=0.003^α^ | p=0.075 | p=0.002^β^ | p<0.001^β^ |
| Percent incorrect | p=0.690 | p=0.321 | p=0.959 | p=0.715 | p=0.251 | p=0.747 | p=0.123 | p=0.189 | p=0.024^α^ |
| Percent anticipatory | p=0.384 | p=0.005^α^ | p=0.006^α^ | p=0.056 | p=0.001^α^ | p=0.005^β^ | p=0.012^α^ | p<0.001^α^ | p<0.001^α^ |
| Percent late | p=0.457 | p=0.139 | p=0.005^β^ | p=0.025^β^ | p=0.001^β^ | p=0.004^α^ | p=0.003^β^ | p<0.001^β^ | p<0.001^β^ |
| Nose-poke discrimination | p=0.632 | p=0.264 | p=0.307 | p=0.789 | p=0.023^β^ | p=0.854 | p=0.004^β^ | p=0.615 | p=0.001^β^ |
| Total number of responses | p=0.042^β^ | p=0.002^β^ | p<0.001^β^ | p=0.001^β^ | p<0.001^β^ | p=0.027^α^ | p<0.001^β^ | p<0.001^β^ | p<0.001^β^ |
| Reaction Time (RT) | p=0.610 | p=0.406 | p=0.003^α^ | p=0.018^β^ | p=0.050^α^ | p=0.019^α^ | p=0.002^α^ | p=0.091 | p=0.543 |

Table S2: Summary of within-subject contrasts for the significant main effect of day during task extinction, showing comparisons to the previous day. ^α^ Indicates a significant increase from the day before whilst ^β^ indicates a significant decrease.
