# Supplementary material for: Instrumental conditioning for food reinforcement in the spontaneously hypertensive rat model of attention deficit hyperactivity disorder
Source: BMC Res Notes. 2017 Oct 30;10:525. doi: 10.1186/s13104-017-2857-5 (PMC5661932; doi:10.1186/s13104-017-2857-5)
Supplement: Supplementary file 3 — Additional file 3: Table S3. Within-subject contrasts for task reversal. Summary of within-subject contrasts for the significant main effect of day during task reversal, showing comparisons to the previous day. α indicates a significant increase from the day before whilst β indicates a significant decrease. [file 13104_2017_2857_MOESM3_ESM.docx]

| Measure | Day 1-2 | Day 2-3 | Day 3-4 | Day 4-5 |
| --- | --- | --- | --- | --- |
| Percent correct | p<0.001^α^ | p<0.001^α^ | p<0.001^α^ | p<0.001^α^ |
| Percent incorrect | p<0.001^β^ | p<0.001^β^ | p<0.001^β^ | p<0.001^β^ |
| Percent late | p<0.001^α^ | p<0.001^α^ | p<0.001^β^ | p<0.001^α^ |
| Nose-poke discrimination | p<0.001^α^ | p<0.001^α^ | p<0.001^α^ | p<0.001^α^ |
| Total number of responses | p<0.001^β^ | p<0.001^β^ | p<0.001^β^ | p=0.004^β^ |
| Reaction Time (RT) | p=0.070 | p=0.003^β^ | p=0.034^α^ | p=0.247 |

Table S3: Summary of within-subject contrasts for the significant main effect of day during task reversal, showing comparisons to the previous day. ^α^ Indicates a significant increase from the day before whilst ^β^ indicates a significant decrease.
